# Discrepancies Between Protocols of Immunization Targeting Internationally Adopted Children in Western Countries

**DOI:** 10.3390/vaccines8010075

**Published:** 2020-02-07

**Authors:** Cecilia Maria Alimenti, Angela Bechini, Sara Boccalini, Paolo Bonanni, Luisa Galli, Elena Chiappini

**Affiliations:** 1 Department of Health Sciences, University of Florence, Viale GB Morgagni 48, 50134 Florence, Italy; angela.bechini@unifi.it (A.B.); sara.boccalini@unifi.it (S.B.); paolo.bonanni@unifi.it (P.B.); 2 Meyer Children’s University Hospital, Department of Health Sciences, University of Florence, Viale Pieraccini 24, 50139 Florence, Italy; luisa.galli@unifi.it (L.G.); elena.chiappini@unifi.it (E.C.)

**Keywords:** vaccination, immunization protocols, internationally adopted children

## Abstract

The immunization status of Internationally Adopted Children (IAC) newly arrived in the adoptive country require a timely assessment and completion of necessary vaccinations. In fact, due to their frequent suboptimal immunization status, IAC are at high risk for vaccine preventable diseases (VPDs). Comparative analysis of immunization protocols adopted in European countries, United States, and Canada disclosed different approaches to the immunization of these children. In order to guarantee the continuity of paediatric immunization schedules that may have been interrupted in countries of origin, a homogeneous and internationally shared standard of immunization in the management of IAC should be provided.

## 1. Introduction

Between 2005 and 2018, receiving countries with the highest number of entries for Internationally Adopted Children (IAC) were United States of America, Italy, Spain, France, Canada, Sweden, Netherlands, Germany, Denmark, Switzerland. In 2018, 4059 children were internationally adopted in United States of America, 1394 in Italy, 658 in Canada, 615 in France, and 445 in Spain [1,2].

A trend towards a reduction was observed starting from 2005. In fact, comparing the number of IAC entered in the United States of America in 2005 (22726), in 2018, only 4059 (–82%) IAC were adopted by the United States of America [1]. 

Globally, in 2018, IAC originated from 10 states: China, India, Colombia, Haiti, Ukraine, South Korea, Vietnam, Bulgaria, Russia, and Ethiopia [1]. 

A large proportion of IAC are susceptible to vaccine preventable diseases (VPDs) because of suboptimal immunization [3,4]. In fact, inefficacy of the vaccines received by the children could be due to inappropriate storage and transport or incorrect administration or interruption of the vaccination schedule [5,6,7]. Moreover, their risk of immunization failure is increased since several other clinical conditions may interfere with immune response to vaccines, including malnutrition or infection with helminths, or human immunodeficiency virus [7]. 

Additionally, vaccines administered in the country of origin may differ from those used in the adoptive country (e.g., monovalent measles vaccine rather than combination measles, mumps, rubella vaccine). Moreover, vaccination schedules may differ by the country of origin [8,9]. 

In order to ensure the continuity of paediatric immunization schedules, the first health evaluation of IAC should include assessment of the immunization status including the assessment of vaccination documentation [5]. 

Vaccine documentation is not always available or, when available, can be incomplete or even false. The vaccine documentation’s poor reliability has already been described in the literature [10]. It is important to make the immunization status of the child adequate without delay because numerous studies have correlated the immunization records poor reliability with IAC unprotection or under-immunization [10,11,12,13].

Written vaccine documentation should only be considered valid if the following criteria are in agreement with the World Health Organization (WHO) schedule: Type of vaccine, age of the child at the time of immunization, date of administration, number of doses, and intervals between doses reported [14,15,16]. 

Even if immunization strategies are still heterogeneous among countries, and sometimes even among regions of the same country (e.g., Italy) [17], pursuing the appropriate vaccination coverage status of IAC is a major challenge for all the health systems. 

According to the WHO, the aim of immunization strategies targeting IAC is to achieve the appropriate immunization status of native-children of the same age [5,18].

Three possible approaches to vaccinating IAC are described in literature to arrive at this aim, but the optimal strategy is still under debate [5,6,19]. The first approach is to re-immunize all adoptees. The second approach is to test antibody titres against VPDs and to re-immunize only those with unprotective antibody titres. The third approach is to assess written immunization records of previous vaccinations and when those are reliable, according to the criteria described before, complete the immunization schedules using an appropriate catch-up schedule based on vaccination records [19]. 

A literature review was conducted to identify which of these strategies were adopted by seven different countries in order to achieve the appropriate immunization coverage against VPDs in newly arrived IAC.

## 2. Materials and Methods 

The immunization strategies adopted in different Western countries were identified by searching guideline websites and national adoption association websites of countries reported to receive the highest number of IAC (United States, Italy, Ireland, France, United Kingdom, Spain, Canada), as previously reported [20]. Moreover, Pubmed was consulted with the search strategy: (International OR abroad) AND (adopted children OR adoption OR adoptees) AND (vaccine OR vaccine preventable disease OR immunization). Reference lists of retrieved articles were analyzed. A comparison among the retrieved protocols was performed focusing on VPD immunization protocols. 

## 3. Results

Protocols adopted in France, Italy, Spain, Ireland, the United Kingdom, the United States, and Canada were retrieved through the search strategy. All the retrieved protocols were national [3,8,17,18,21,22]. The English and Spanish protocols were published in 2004 and 2008, whereas the other five were published between 2013 and 2019. Table 1 and Table 2 summarize the protocols recommendations. Moreover, the European Centre for Diseases Prevention and Control (ECDC) health guidance on vaccination was considered, even if it focused on newly arrived adults and children migrants [21].

### Summary of Findings

All the reviewed protocols recommend achieving the appropriate immunization coverage promptly after IAC arrive in the new country. The immunization coverage considered as appropriate is the same for all protocols: In fact, the immunological target to be achieved is an immunological coverage similar to one of the native children of the same age [3,8,17,18,22,23]. This goal should be achieved as quickly as possible after IAC arrival. 

Three possible approaches to determine the appropriate immunization coverage for IAC are described in the literature, but the optimal strategy is still under debate. These approaches are adopted differently for different vaccines by the different countries [5,6,19].

The US protocol identified some important questions that have to be carefully considered when choosing one of these approaches, including the urgency of school entry [8].

The assessment of the vaccination records of the country of origin should be accurate, following the WHO instructions above described [14,15,16]. 

Some indicators of reliability of the vaccination records were described in a few protocols. Comparison of serological results for hepatitis B (HBV) and vaccination for HBV reported in the immunization record can be a way to assess the reliability of the child’s documentation. Another additional way to investigate the reliability of the immunization records is to check for a scar when the Bacille Calmette Guerin (BCG) is reported in the documentation [8,18,22].

All protocols recommend performing serological testing to assess the IAC status against HBV. It allows to identify a previous infection, ongoing infection, protective status, or susceptibility. An interesting difference among protocols concern the fact that only some experts recommend testing for the hepatitis B core antibody (HBcAb). In fact, the Italian, the French, and the Canadian protocols suggest only testing for hepatitis B surface antigen (HBsAg) and; hepatitis B surface antibody (HBsAb) [3,17,23], while the American, the English, the Spanish, the Irish, and the ECDC protocols recommend also HBcAb serotesting. Testing for HBcAb makes the HBV-assessment more complete. In fact, positive HBcAb and negative HBsAg can lead to four different conditions: Acute HBV infection, very low level of anti-HBs in the serum that are not detectable by serotesting, susceptibility to HBV because of a false positive anti-HBc, undetectable level of HBsAg present in serum when the child is actually chronically infected [8].

Except as already mentioned for hepatitis B according to the different protocols selected cases should be tested for diphtheria and tetanus (DT), measles-mumps-rubella (MMR), haemophilus influenzae type b (HiB), and poliovirus antibodies (Table 1).

As far as diphtheria tetanus and pertussis vaccine is concerned (DTP), the Italian protocol recommends testing for tetanus antibody titres only for children aged younger than seven years of age wherever their documentation of vaccination is unavailable or incomplete. An antibody titre for tetanus >/= 0.1 UI/ML is considered protective, and moreover, is also considered the proof of protection against diphtheria (as a surrogate of diphtheria seropositivity) [17].

In the French protocol, tetanus serology is recommended one month after the administration of one dose of the vaccine, in order to detect if the booster reaction takes place in those IAC who had probably received DTP vaccination in their country of origin [23].

In the Spanish protocol, serology is requested when: More than three doses of DTP are reported in the documentation; immunization records are unreliable; severe local reaction takes place at the first dose of vaccine; to evaluate a booster reaction after a first dose of vaccine [18].

In the US AAP protocol, serology test for diphtheria and tetanus are requested for IAC under six months of age with or without written documentation of immunization.

In the US AAP protocol, Hib serological test is recommended only in IAC under five years of age with immunization records available to check titres to validate. Concerning hepatitis A (HVA), the Italian, the US, and the ECDC protocols suggest testing serologically for IgG and IgM against HVA [8,17,21].

The approach of performing systematically the serology for all VPDs is considered by most protocols as expensive, not always available, and not applicable to all vaccinations [17,18]. Moreover, antibody titres do not always confirm whether the child is fully immunized, e.g., no absolute serological correlates of protection exist for measles, mumps, and rubella [24]. In fact, for these VPDs (even if it seems that high levels of circulating antibodies are important in protecting against outbreaks) current data does not give conclusive evidence of what level of antibodies should be considered protective for each of the VPDs considered.

Most protocols support the re-vaccination of IACs when previous vaccination documentation is unavailable or considered to be unreliable [8,18]. In fact, vaccination of already immune children is safe [8,18]. Repeating some vaccinations can lead to minimal risks of adverse events, especially for diphtheria-tetanus-pertussis toxoids, but above all it avoids the considerable risk of inadequate immunization [22].

Regarding anti-Hib vaccines, anti-meningococcal C, conjugate anti-pneumococcal, and varicella vaccine, these vaccines are usually not administered in IAC in their country of origin. Thus, they should be administered, following the National schedule [8,18] (Table 2). 

## 4. Discussion

This paper focuses on different protocols for immunizing newly arrived IAC against vaccine preventable diseases (VPDs). At the best of our knowledge, this is the first study comparing existing protocols for the immunization of IAC in Western countries. 

All protocols were provided on a national level. Even if the target of immunization of IAC is similar for all protocols (to obtain the same immunological status of native children), there is no homogeneous and internationally shared standard of care for the management of IAC.

Regarding the retrieved protocols, many factors may justify the discrepancies. These documents have been published in different periods, with the English and Spanish protocols being produced in 2004 and 2008. Therefore, new evidence may have been included in the protocols produced more recently by Italy, US, France, and Canada. 

Another possible reason for discrepancies among protocols is that, to date, there is conclusive data which shows the best strategy among re-immunizing all IAC, performing serology and immunizing based on serological results, or immunizing considering immunization documentation.

Few sparse data reported in the literature suggests that the reliability of vaccine documentation may vary by country of origin [11,24,25]. However, such poor data may depend on the region of the country mainly represented in the specific dataset of the study and is insufficient to generalize results of an entire country. Moreover, none of the retrieved protocols based the adopted strategy on the child’s origins. More data on this matter would be helpful and we hope that our review may stimulate further research on this topic. Recently, the ECDC has produced guidance on screening and vaccination for infectious diseases in newly arrived migrants within the European Union [21].

This document is a useful instrument but has the limitation of dealing with both adults and children and it does not focus specifically on IAC that are a high risk, peculiar population. A systemic approach categorized per country of origin and age may be useful.

A possible limitation of this study is that some protocols may have been missed by our research.

Another limitation is that the specifics of vaccine composition were not analyzed, since they were not included in most protocols. Several differences can be present across countries but from a practical point of view, this can only marginally affect the protocol.

## 5. Conclusions

In conclusion, since screening protocols used in various countries are not homogeneous, the introduction of an optimized internationally shared protocol focusing on IAC would be highly desirable.

## Figures and Tables

**Table 1:** Serology tests recommended by different western countries to assess the immunization status of newly arrived Internationally Adopted Children (IAC).

	Refers to	Year	Country Protocol:	HBV	DTP	Hib	POLIO	MMR-V	HAV
SEROLOGY TESTS RECOMMENDED FOR	IAC + Imm.	2013	ITALY	HBsAg, HBsAb	T ^1^				IgG + IgM
	IAC + Imm.	2016	IRELAND	HBsAg, HBsAb, HBcAb				R, V ^7^	
	IAC	2004	UK	HBsAg, HBsAb, HBcAb					
	IAC + Imm.	2014	FRANCE	HBsAg, HBsAb	T ^2^			V ^8^	
	IAC	2008	SPAIN	HBsAg, HBsAb, HBcAb	T, D ^3^			Me, R ^9^	
	IAC + Imm.	2018	CANADA	HBsAg, HBsAb				V ^10^	
	IAC	2019	US AAP	HBsAg, HBsAb, HBcAb	T, D ^4^	Hib ^5^	P1,2,3ab ^6^	MMR-V ^11^	IgG + IgM ^12^
	IAC	2019	US CDC	HBsAg, HBsAb, HBcAb	T, D^14^	Hib^14^		MMR-V ^14^	IgG + IgM ^12^
	Imm.	2018	ECDC	HBsAg, HBsAb, HBcAb ^13^					

IAC: Internationally adopted children; Imm.: Immigrants; UK: United Kingdom; HBsAg: Hepatitis B surface antigen; HBsAb: Hepatitis B surface antibody; HBcAb: Hepatitis B core antibody; T: Tetanus serology test; D: Diphtheria; P: Pertussis; Hib: Type B *Haemophilus Influenzae*; P1,2,3ab: Type 1, 2, and 3 poliovirus; MMR-V: Measles-mumps-rubella-varicella vaccine; R: Rubella, V: Varicella; Me: Measles; Mu: Mumps; HAV: Hepatitis A. ^1^ In the Italian protocol, tetanus serology is recommended in children younger than seven years of age with uncertain or unavailable documentation; ^2^ in the French protocol, tetanus serology is recommended one month after the administration of one dose of the vaccine, in order to detect if booster reaction takes place in those IAC who had probably received Diphtheria-Tetanus-Pertussis (DTP) vaccination in their country of origin; ^3^ in the Spanish protocol, serology is requested when: More than three doses of DTP are reported in the documentation; immunization records are unreliable; severe local reaction takes place at the first dose of vaccine; to evaluate a booster reaction after a first dose of vaccine; ^4^ in the US AAP protocol, serology test for diphtheria and tetanus are requested for IAC under six months of age with or without written documentation of immunization; ^5^ in the US AAP protocol, Hib serological test is recommended only in IAC under five years of age with immunization records available to check titres to validate; ^6^ in the US AAP protocol, to confirm poliovirus immunity it is suggested to test the neutralizing antibody for types 1, 2, and 3; ^7^ in the Irish protocol, varicella and rubella serologies are recommended in girls of childbearing age (guidelines 2015); ^8^ in the French protocol, varicella serology is recommended in newly arrived older children and adolescents; ^9^ in the Spanish protocol, serological tests for measles and rubella are required if documentation is unreliable; ^10^ in the Canadian protocol, varicella serology (VZV IgG) is recommended in IAC older than 13 years; ^11^ in the US AAP protocol, MMR-V serological tests are suggested in children older than 12 months. Measles antibody testing may be irrelevant if the child has not received mumps or rubella vaccines and needs the MMR vaccine anyway. The best indication of immunity to varicella is the documented receipt of two doses of the varicella vaccine; ^12^ in the US protocols and in the Italian protocol, serological tests for hepatitis A virus (IgG and IgM anti-HAV) is recommended; ^13^ in the ECDC guidelines, diagnostic testing to assess hepatitis B are recommended in migrants arriving from intermediate (>2%) to high (>5%) prevalence countries; ^14^ in the US CDC protocol for children >/= 6 months of age, testing can be done for diphtheria (IgG), tetanus, (IgG) hepatitis B (as outlined above) and Hib. For children >/= 12 months of age, testing can be done for measles, mumps, rubella, hepatitis A, and varicella.

**Table 2:** Vaccinations recommended by different Western countries for newly arrived Internationally Adopted Children (IAC).

	Refers to	Year	Country Protocol:	DTaP/IPV/Hib/HBV	MMR-V	Men	PCV	BCG	HPV	Rotavirus
RECOMMENDED VACCINE FOR	IAC + Imm.	2013	ITALY	Vaccinate all IAC using age appropriate vaccination and follow the national schedule						
	IAC + Imm.	2016	IRELAND	Three doses for IAC from four months to <18 years of age ^1^	12months-18 years of age: Two doses ^2^	One dose +/- a booster	4–12 months two doses two months apart;IAC 1 yr-2 yrs: one dose. NO if IAC >2 years of age	One dose (All IAC from four months-15 yrs of age); one dose high risk 15–18 yrs	Guidelines 2015: vaccinate all females 12–13 years old	Not reported in this guidance
	IAC	2004	UNITED KINDOM	IAC from two months of age up to 12 months with 6-in-1 vaccine ^2^	>13-15 months: Two doses ^2^	Three, two, or one dose depending on IAC age	IAC>2 years only if splenic dysfunction is known	Vaccine IAC <14 years	Not reported in this guidance	Not reported in this guidance
	IAC + Migrants	2014	FRANCE	No Hib if older than five years of age. HBV IAC < 11 years of age: Three doses, 11–15 yrs 2/3 dose schedule	Two doses ^3^	MenC IAC: 12–24 months: One dose + one booster	Two doses +/- a booster Depending on IAC age	Vaccinate IAC older than three months with Tuberculin skin test -	Not reported in this guidance	Not reported in this guidance
	IAC	2008	SPAIN	No Hib if older than five years of age	Vaccinate IAC >12 months old	Vaccinate IAC with age appropriate schedule ^4^	<6 years of age	Not reported in this guidance	Not reported in this guidance	Not reported in this guidance
	IAC + Migrants	2018	CANADA	Vaccinate all IAC with missing or uncertain vaccination records	Two doses: First dose 12–15 months of Age; a booster at 18 months or anytime later.^5^	Vaccinate all IAC with missing or uncertain vaccination records	Vaccinate all IAC with missing or uncertain vaccination records	Vaccine children living in very specific geographic areas	Vaccinate all IAC with missing or uncertain vaccination records	Vaccinate all IAC with missing or uncertain vaccination records
	IAC	2019	US AAP	Immunize following AAP catch-up schedule			=	=	=	=
	IAC	2019	US CDC	Immunize following ACIP schedule^6^			=	=	=	=
	Migrants	2018	ECDC	Offer DTaP/IPV/Hib vaccine to all migrants without documentation records; HBV: Offer to all migrants from intermediate to high prevalence countries who do not have evidence of vaccination or immunity	Vaccinate all IAC without documentation records.	Not reported in this guidance	Not reported in this guidance	Not reported in this guidance	Not reported in this guidance	Not reported in this guidance

Note: IAC: Internationally adopted children; Imm.: Immigrants; UK: United Kingdom; HBsAg: Hepatitis B surface antigen; HBsAb: Hepatitis B surface antibody; HBcAb: Hepatitis B core antibody; T: Tetanus; D: Diphtheria; DTaP Diphtheria-Tetanus-acellular-Pertussis; Hib: Type B *Haemophilus Influenzae*; IPV inactivated poliovirus; MMR-V: Measles-mumps-rubella-varicella vaccine; R: Rubella, V: Varicella; Me: Measles; Mu: Mumps; HAV: Hepatitis A; PCV: Pneumococcal conjugate vaccine; Men: meningococcal vaccine; BCG: Bacille Calmette Guerin; yrs: Years. ^1^ IRELAND: Vaccinate IAC from four months to <10 years with 6-in-1 vaccine three doses two months apart for IAC from four months to <10 years of age; vaccinate with Tdap/IPV IAC from 10 years to <18 years of age: Three doses two months apart. ^2^ UK: Vaccinate IAC from two months of age up to their first birthday with 6-in-1 vaccine: Three doses one month apart; use DTaP/IPV/Hib three doses one month apart for IAC from one to 10 years of age. ^2^ IRELAND schedule depending on age: 12 months–4 yrs: First dose, 4–5 yrs second dose. IAC from 4 to <18 years of age: Two doses MMR at one-month intervals. Offer varicella vaccine to girls of childbearing age and some immunocompromised children, e.g., those with HIV infection. ^2^ UK: One dose of MMR vaccine is recommended in IAC 13–15 months and a booster at 3–5 years; IAC at 1–5 years: First dose on arrival and second dose either three months later or with pre-school booster, as appropriate; IAC >5 years: Two doses separated by three months; varicella NOT specified. ^3^ France: Vaccinate all IAC between two years and 16 years of age, with MMR vaccine (two doses one month apart). Vaccinate adolescent IAC (12–16 years of age) with varicella vaccine: Two doses, one month apart. ^4^ SPAIN Men: the Spanish protocol recommend vaccinate IAC following the National schedule^5^ Canada: The first dose of a combination MMR or MMRV vaccine is given between 12 and 15 months of age, with a booster at 18 months or anytime later, though typically before school entry. Vaccinate against varicella following age-appropriate schedule for all IAC. ^6^ CDC immunization should be given according to the current ACIP schedule for catch-up vaccination and if there is uncertainty regarding immunization status or validity of the immunization records, the child should be immunized according to the ACIP schedule. UK MenC: Depending on age: MenC: IAC< 5 months: Three doses; 5–12 months: Two doses; 1–5 years: Single dose; > 5 years: Single dose. PCV FRANCE: IAC from 2–6 months old two doses and a booster (2,4,11 months); IAC 7–11 months old: Two doses two months apart and a booster after one year; IAC 12–23 months old two doses two months apart. BCG IRELAND: One dose of BCG vaccine should be given to low risk children from four months up to 15 years of age and high-risk children and adults up to 35 years of age. BCG UK recommends providing BCG vaccination to IAC aged <14 year.

## References

[1] Selman P. (2019). Global Statistics for Intercountry Adoption: Receiving States and States of Origin 2005–2018. https://assets.hcch.net/docs/a8fe9f19-23e6-40c2-855e-388e112bf1f5.pdf.

[2] Commission for Intercountry Adoptions Data and perspectives in Intercountry Adoptions. Summary Report Dossiers From 2018–2019. http://www.commissioneadozioni.it.

[3] Dixit D., Rajapakse N., Kuhn S. (2018). Caring for Kids New to Canada Immunizations: Bringing Newcomer Children Up-to-Date. https://www.kidsnewtocanada.ca/screening/immunizations..

[4] Giordano D., Provenzano S., Santangelo O., Piazza D., Ferraro D. (2018). Active immunization status against measles, mumps, rubella, hepatitis B in internationally adopted children, surveyed at the university hospital of Palermo, Sicily. Ann. Ig..

[5] Schulte J.M., Maloney S., Aronson J., Gabriel P.S., Zhou J., Saiman L. (2002). Evaluating acceptability and completeness of overseas immunization records of internationally adopted children. Pediatrics.

[6] Centers for Disease Control and Prevention. https://wwwnc.cdc.gov/travel/yellowbook/2020/family-travel/international-adoption.

[7] Sollai S., Ghetti F., Bianchi L., De Martino M., Galli L., Chiappini E. (2017). Infectious diseases prevalence, vaccination coverage, and diagnostic challenges in a population of internationally adopted children referred to a Tertiary Care Children’s Hospital from 2009 to 2015. Medicine.

[8] Staat M.S. International Adoption: Immunization Considerations. https://www.uptodate.com/contents/international-adoption-immunization-considerations.

[9] (2019). Monitoring Immunization.

[10] Jones V.F., Schulte E.E., Care A.C.O.F. (2019). Comprehensive Health Evaluation of the Newly Adopted Child. Pediatrics.

[11] Schulpen T., Van Seventer A., Rümke H., Van Loon A. (2001). Immunisation status of children adopted from China. Lancet.

[12] Miller L., Ake J.A., Jelacic S., Ciol M.A., Watkins S.L., Murray K.F., Christie D.L., Klein E.J., Tarr P.I. (2005). Health of Children Adopted From Guatemala: Comparison of Orphanage and Foster Care. Pediatrics.

[13] Viviano E., Cataldo F., Accomando S., Firenze A., Valenti R., Romano N. (2006). Immunization status of internationally adopted children in Italy. Vaccine.

[14] (2019). Vaccine-Preventable Diseases: Morning System. http://apps.who.int/immunization_monitoring/globalsummary/diseases..

[15] Verla-Tebit E., Zhu X., Holsinger E., Mandalakas A.M. (2009). Predictive Value of Immunization Records and Risk Factors for Immunization Failure in Internationally Adopted Children. Arch. Pediatr. Adolesc. Med..

[16] Staat M.A., Stadler L.P., Donauer S., Trehan I., Rice M., Salisbury S. (2010). Serologic testing to verify the immune status of internationally adopted children against vaccine preventable diseases. Vaccine.

[17] The Italian Pediatric Society News Directives for the Healthcare of Migrant Children. Proceedings of the Consensus Conference GLNBI-SIP.

[18] Federación CORA-Coordinadora de Asociaciones en Defensa de la Adopción y el Acogimiento (2008). Pediatric Guidelines for Adoption.

[19] Pickering L.K., Baker C.J., Freed G.L., Gall S.A., Grogg S.E., Poland G.A., Rodewald L.E., Schaffner W., Stinchfield P., Tan L. (2009). Immunization Programs for Infants, Children, Adolescents, and Adults: Clinical Practice Guidelines by the Infectious Diseases Society of America. Clin. Infect. Dis..

[20] Chiappini E., Bortone B., Borgi S., Sollai S., Matucci T., Galli L., De Martino M. (2019). Infectious Diseases in Internationally Adopted Children and Intercountry Discrepancies Among Screening Protocols, A Narrative Review. Front. Pediatr..

[21] British Association for Adoption and Fostering (2008). Health Screening of Children Adopted from Abroad.

[22] De Monléon J., Regnier F., Ajana F., Baptiste C., Callamand P. (2014). Mise à jour des vaccinations de l’enfant arrivant de l’étranger (adopté, réfugié ou migrant) en France Catch-up vaccination of worldwide newcoming (adopted, refugee or migrant) children in France. Arch. Pediatr..

[23] European Centre for Disease Prevention and Control Public Health Guidance on Screening and Vaccination for Infectious Diseases in Newly Arrived Migrants within the EU/EEA. https://www.ecdc.europa.eu/en/publications-data/public-health-guidance-screening-and-vaccination-infectious-diseases-newly.

[24] Woudenberg T., Van Binnendijk R., Veldhuijzen I., Woonink F., Ruijs H., Van Der Klis F., Kerkhof J., De Melker H., De Swart R., Hahné S. (2019). Additional Evidence on Serological Correlates of Protection against Measles: An Observational Cohort Study among Once Vaccinated Children Exposed to Measles. Vaccines.

[25] Venturini E., Piccini P., Tersigni C., Chiappini E., Galli L. (2019). Systematic review shows that immunising internationally adopted children is a major challenge for primary health care. Acta Paediatr..

